# Lignin peroxidase ligand access channel dysfunction in the presence of atrazine

**DOI:** 10.1038/s41598-018-24478-w

**Published:** 2018-04-16

**Authors:** János Ecker, László Fülöp

**Affiliations:** Szent István University, Department of Chemistry, 2100 Gödöllő, Hungary

## Abstract

Studies have determined that the white-rot basidiomycete *Phanerochaete chrysosporium* is capable of biodegrading the atrazine herbicide with its broad-specificity enzymes, but the particular role of biocatalysts is still unclear. In the case of lignin peroxidase, a ligand access channel connected to the active heme cofactor provides access to the active site for potential small-sized substrates. Experimental results show that lignin peroxidase is unable to degrade atrazine, therefore, the primary goal was to determine whether there is any connection between the structural and dynamical properties of the enzyme and its incapability to degrade atrazine. The results of protein-ligand docking and molecular dynamics study correlate with relevant, published NMR and molecular dynamics data, and give the answer to the lack of atrazine degradation by lignin peroxidase which has already been established by numerous authors using experimental methods. Atrazine has no access to heme edge due to the electric charges of the delocalized s-triazine ring. The detected phenomenon suggests that the small size of the ligands only is not a sufficient condition to access the active site. Their physicochemical properties influence the structural behaviour of the channel.

## Introduction

In many countries with intensive agriculture, the photosystem II-inhibitor atrazine (1-chloro-3-ethylamino-5-isopropylamino-2,4,6-triazine) is one of the most widely used herbicide by farmers, in combination with other active ingredients. More than fifty plant species are treated with atrazine including citrus, corn, soybeans, sugar cane, vines and forest species. Several environmental and ecological problems have been identified in relation to atrazine, so its biodegradation is an extensively researched subject. The decrease in atrazine concentration in the soils is the result of chemical, physical and biological processes. Atrazine may be bound to various (organic or inorganic) soil components according to a number of mechanisms, and some results suggest that bonds with variable strengths are formed. Multiple binding sites exist, but these are different in their individual bond strengths. Atrazine, like other pesticides, can persist only in the high-energy sites (retention)^1^, otherwise, it desorbs and becomes biodegradable^2^.

Atrazine is a very weak base. It can be said that the atrazine adsorption in soils at an acidic pH is much more explicit than in neutral or alkaline pH^3–7^. Furthermore, hydrolysis of atrazine under acidic conditions is faster than under neutral conditions, and the reaction product is hydroxyatrazine^8^.

Many microorganisms were studied for their abilities to metabolize atrazine including members of the genera *Pseudomonas*, *Acinetobacter*, *Agrobacterium*, *Arthrobacter*, *Rastonia and Nocardioides*^9–13^. In some studies, soil fungi were found to be dominantly causing the dealkylation of atrazine while bacteria were responsible for its further degradation and mineralization^14^. Many fungi belonging to genera such as *Aspergillus*, *Rhizopus*, *Fusarium*, *Penicillium*, *Trichoderma* and *Phanerochaete* have been found to be capable of degrading atrazine^15,16^.

The *Phanerochaete chrysosporium* (*Basidiomycota*) can degrade several polluting compounds (dioxins, polychlorinated biphenyls) with its broad-specificity enzymes: lignin peroxidase, manganese-dependent peroxidase and laccase^17–19^. Lignin degradation is a widely researched subject^20^, and how these three enzymes interact with their ultimate substrate, lignin, has been intensely investigated. Lignin peroxidase may be involved in the oxidation of the soluble, partially degraded lignin fragments, however, the possibility that lignin peroxidase can directly oxidize lignin in the partially degraded plant cell walls cannot be excluded^21^. Lignin peroxidase has a so-called ligand access channel which allows direct interaction between the substrate and the heme. Residues His82, Ile85, Glu146, Phe148, Asp183, Val184 and Gln222 are located in this channel. The channel is sterically restricted and does not allow access to large bulky (lignin) substrates^21^. Two conserved histidines, the proximal (His177), distal (His48) and the distal side arginine (Arg44) residues are conserved for peroxidative catalytic function, similarly to the class I and class III heme peroxidases^22^. For the high-redox potential substrates, such as veratryl alcohol (3,4-dimethoxybenzylalcohol, veratrol), the oxidation site in lignin peroxidase is localized on the enzyme surface at a catalytically active tryptophan (Trp171)^23^. A second substrate oxidation site is located at the heme edge, near to a surface-exposed Glu146^24^.

The dynamic properties of lignin peroxidase show that the enzyme’s natural substrate, veratrol, can easily approach heme through the ligand access channel. The analysis of molecular dynamics (MD) results also showed significant fluctuations of the channel^25^, where the open and closed states of the ligand access channel are in constant equilibrium in solution. This phenomenon is a characteristics of many enzymes (e.g. the structurally very similar cytochrome C peroxidase). NMR experiments confirm that the ligand can bind to the open conformer, and has access to the active site, then the process of binding shifts the equilibrium towards the open conformer^26^.

In a classic liquid culture experiment, *P*. *chrysosporium* was able to degrade atrazine, through oxidative N-dealkylation. The fungus cleaved the ethyl group, but was not able to open the heterocyclic ring. However, the biotransformation rate of atrazine was not significant, and the experiment has also failed to demonstrate that lignin peroxidase contributed to the process in any way^16^. Another important experimental result showed that purified peroxidases alone were not at all able to degrade atrazine, as neither metabolites nor carbon dioxide were detected^27^. Significant amounts of Mn^2+^ ions repressed the production of lignin peroxidase, and stimulated the expression of manganese peroxidase nearly tenfold^28^.

The full sequence and structure of lignin peroxidase are known. The enzyme is a homodimer consisting of two subunits of 351 amino acids. Each subunit contains two Ca^2+^ ion binding sites, and the secondary structures are mostly α-helices^29^.

## Methods

The three-dimensional model of atrazine and the energy minimization of the structure was made by Avogadro^30^ (v1.1.1). The docking procedure was carried out with the DockingServer^31^ web-based application, and the results were analyzed with AutoDockTools^32^ (v1.5.6). The applied enzyme structure was obtained from the RCSB PDB database (PDB: 1B82) with a resolution of 1.8 Å. The structure contained a non-relevant R114A mutation^29^. MD simulations of lignin peroxidase were performed with NAMD software^33^ (v2.12) using the CHARMM36 force field^34^. The CHARMM-compatible parameters for atrazine were calculated with the Force Field Toolkit plugin^35^ (v1.1) of VMD software^36^ (v1.9.3) adding previously generated parameters based on quantum chemical calculations^37^. The images were rendered with Tachyon Parallel/Multiprocessor Ray Tracer^38^ and AutoDockTools.

Histidines were protonated (HSD form; Histidine with hydrogen on the delta nitrogen), the enzyme structure (with heme and two Ca^2+^ ions) was solvated, neutralized, and the NaCl concentration was set to 0.15 mol dm^−3^. Each of the three minimizations were run for 30,000 steps with Conjugate Gradients method. The temperature was set to 288 K, 298 K and 308 K, respectively, to create three different conformers. Then MD analysis was performed on every minimized structure for 10 ns. The NPT ensemble (constant number of atoms, pressure and temperature) was employed with Periodic Boundary Conditions and Particle Mesh Ewald electrostatics with a steady pressure of 1 bar. From each of the 10-ns-long trajectories, a structure with open state of the ligand access channel was chosen. These conformers were the input structures of the docking procedure.

Only chain A of the lignin peroxidase was used (the two chains are identical) without solvent. The centre of the simulation box was the His82 residue which is the entrance of the ligand access channel, and the size of the box was set to 20 * 20 * 20 Å. The partial atomic charges of lignin peroxidase and atrazine were calculated with the Gasteiger method^39,40^. For every lignin peroxidase conformer, the whole docking process contained 255 docking trajectories. During the process, the lignin peroxidase structure remained rigid; only the atrazine was flexible. The docking result with highest frequency value was chosen, and the lignin peroxidase-atrazine complex was solvated and minimized under the same conditions. The complex was refined with a 5-ns-long MD simulation with the same temperature value and settings used during the creation of the given conformer in order to study its stability. The binding free energies were calculated in solvated phase with NAMDEnergy plugin. The binding free energies contained van der Waals, electrostatic, polar and non-polar contributions^41^. Finally, a 100-ns-long MD simulation was performed in addition to the 5 ns refinement with NVT ensemble (constant number of atoms, volume and temperature) to collect more information about atrazine’s behaviour in a larger timescale.

### Data availability

The datasets generated during and/or analysed during the current study are available in the [Mendeley Data] repository, [https://data.mendeley.com/datasets/dvjms8448j/draft?a = 8086c15c-10e7–4f80-8d69-7004099ac95a] and [https://data.mendeley.com/datasets/b2wn6fdcj4/draft?a = 67f29979-7939-459e-a4a5-2a46f69466b6].

### Equipment and settings

- Rendering: Tachyon Parallel/Multiprocessor Ray Tracer (VMD software plugin)
- Final format: GIMP 2.8, compression: LZW

## Results and Discussion

### Evaluation of docking results

**Figure 1:**
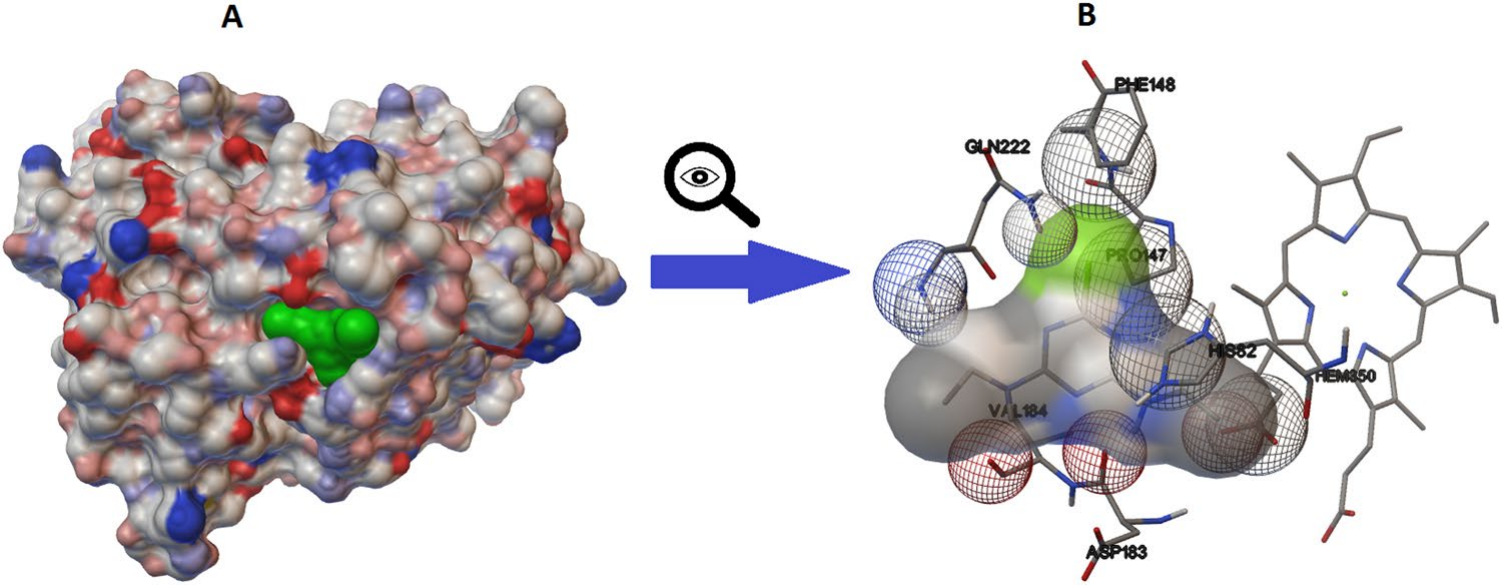
The structure of the [298 K] lignin peroxidase-atrazine complex after the docking procedure. Complex (**A**) is visualized by the molecular surface of its components. Partial charges of the lignin peroxidase residues are highlighted in red (negative) and blue (positive), the incorporated atrazine is highlighted in green. The interacting side chains (**B**) are represented by stick models; wireframe spheres mark the residue atoms involved in the interaction. The size of the spheres is determined by their distance from the reader’s eyes.

The energetics data of three lignin peroxidase-atrazine complexes are summarized in Table 1. Negative values indicate energy release which is thermodynamically favourable in that the atrazine incorporated into the environment of His82 (Fig. 1). For every conformer, the binding of the atrazine to the target site of lignin peroxidase is thermodynamically favourable, but the frequency value is only 16% and 10% at temperatures of 288 K and 308 K, respectively. The possibility of the formation of these complexes is very low. The frequency is 53% in the case of the lignin peroxidase conformer created at 298 K, therefore, this was the input structure of the 5 ns refinement process, to study the stability of the complex and the atrazine’s interactions with the ligand channel residues.

**Table 1:** Summarized data of the best ranked atrazine-lignin peroxidase docking result.

Temperature	Est. Free Energy of Binding	vdW + Hbond + desolv. Energy	Electrostatic Energy	Total Intermolec. Energy	Frequency	Interact. Surface
288	−5.55	−6.30	+0.02	−6.29	16%	552.103
**298**	**−5**.**54**	**−6**.**07**	**−0**.**05**	**−6**.**12**	**53%**	**575**.**72**
308	−5.40	−5.93	−0.03	−5.96	10%	541.415

Temperatures are in K, energies in kcal mol^−1^. Frequency is a ratio of the dockings resulting in the same geometry and can be considered as a probability of the given result. High frequency suggests reliable result and specific binding.

Protein-ligand interactions between atrazine and the amino acids of the interacting side chains of lignin peroxidase fall into the following categories: hydrogen bond, polar, hydrophobic, pi-pi and other (Table 2). The Cα carbonyl group of Asp183 come into contact through H-bonds with the isopropylamine nitrogen of atrazine. There is also an undefined contact between the oxygen of the Cα carbonyl group and the number 1 nitrogen of the s-triazine ring. The distance between these two atoms is only 2.68 Å. Since there is no direct contact between the nitrogen and oxygen, and both atoms have negative partial atomic charges, therefore, this state is energetically unfavourable, and in a fully dynamic system, it cannot emerge.

**Table 2:** Decomposed interaction energies of the [298 K] atrazine-lignin peroxidase docking in kcal mol^−1^.

Hydrogen bonds	Polar	Hydrophobic	Other^b^
GLN222^a^ (−1.1768)	HIS82^a^ (−0.6902)	PRO147 (−0.7052)	ASN221 (−0.8759)
VAL184^a^ (−1.1032)		ILE85^a^ (−0.1785)	PHE148^a^ (−0.2982)
ASP183^a^ (1.8798)			

^a^The residue is part of the ligand access channel. ^b^The type of the interactions is undefined.

### Structural stability of the docking complex

**Figure 2:**
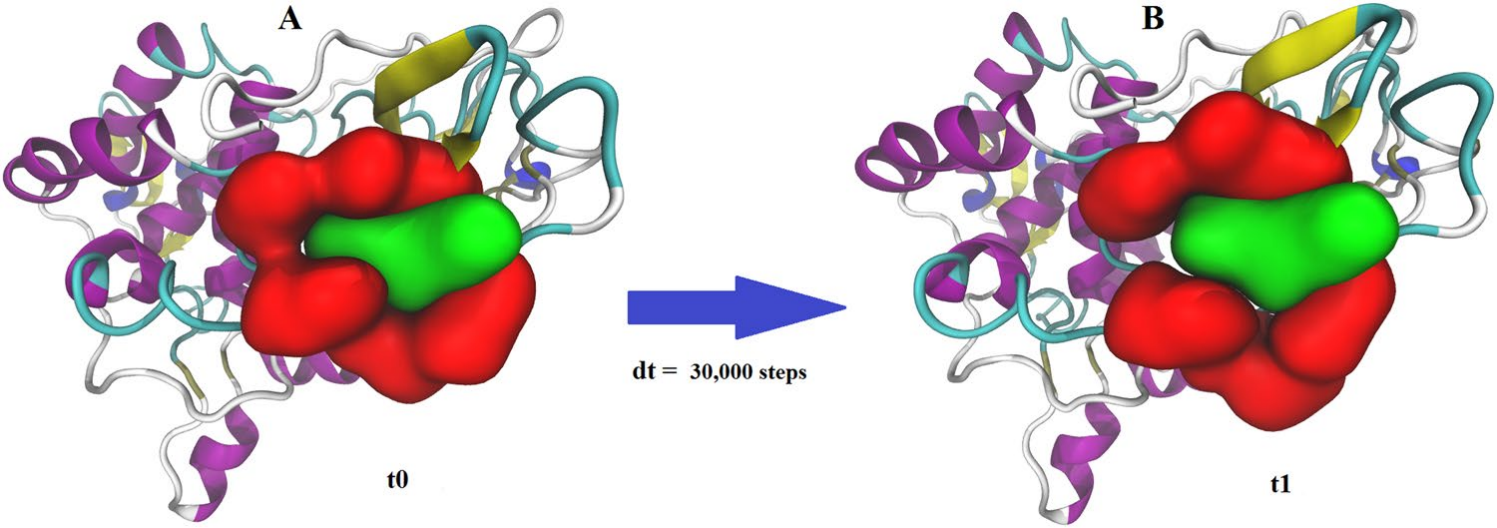
State of the docking structure (**A**) and the structure of the [298 K] complex after a minimization of 30,000 steps without the solvent (**B**). The residues forming the access channel and the atrazine are coloured in red and green with QuickSurf representation. The rest of the enzyme is represented with a NewCartoon drawing method and the coloring method based on secondary structure. α-helices are coloured in purple, β-strands in yellow, 3–10 helices in blue, turns in cyan and coils in white. The resolution of the ligand channel residues-atrazine segment was intentionally reduced to emphasize the structural difference between the t0 and t1 state, and the residues have not been labeled for clarity.

The Cα root mean square deviation (RMSD) of the ligand channel residues and the atrazine minimization showed that the initial structure, the [298 K] docking complex, was not in an optimal state. The 30,000 steps were enough for the complex to reach a relaxed state. The minimum RMSD value was 0.040 Å, the maximum was 1.490 Å. The system reached the maximum value in 29.55 ps, and after approximately 20 ps, the system was in a relaxed state. Since the lignin peroxidase was completely rigid during the docking procedure, the structural difference between the docking and the minimized complex (Fig. 2) indicates that in a fully dynamic system the t0 state is unfavourable. The t1 state is a relaxed one, but since the t1 complex is a result of energy minimization where the atoms have no kinetic energy, conclusions can only be drawn from the MD results.

### Behaviour of the ligand access channel in the presence of atrazine

The fluctuation and deformations of the lignin peroxidase ligand channel residues in aqueous solution is a known phenomenon described by Gerini and her colleagues (2003)^25^. The access channel shows remarkable distortions from the crystal structure (Supplementary Fig. S1) even during a 150-ps-long MD simulation. These rapid conformational changes of the ligand channel residues are typical of lignin peroxidase when a potential substrate or a small molecule is not present in the environment of the channel. In the presence of certain molecules (e.g. veratrol which is a natural substrate of the lignin peroxidase enzyme), the observed phenomena may be different according to the established interactions between the ligand and the ligand channel residues^25^.

**Figure 3:**
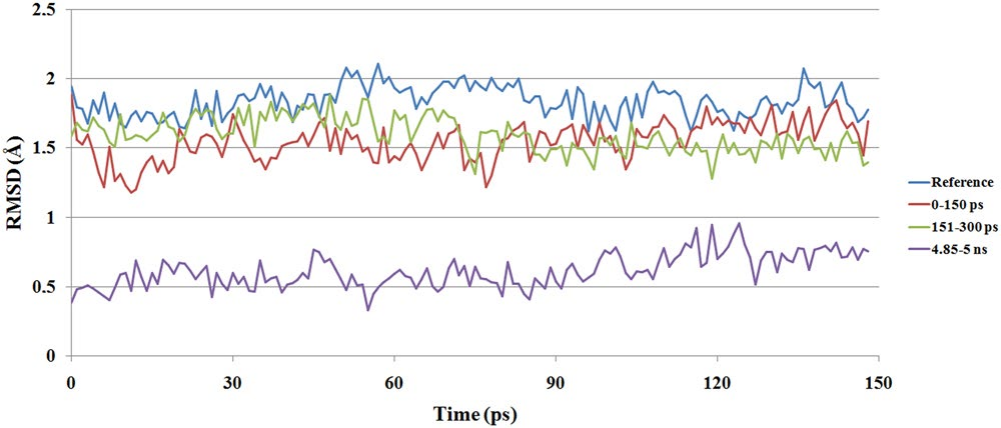
Fluctuation of the ligand channel in the presence of atrazine on various timescales. The reference (blue) was the 150-ps-long MD simulation of lignin peroxidase without atrazine. The fluctuation was clearly detectable during 150 ps MD. The other structure which contained atrazine shows less significant fluctuations even during the first 150 ps and only mild fluctuations during the rest of the 5 ns MD. The 0–150 (red) and the 151–300 (green) ps intervals show only a minimal decrease in channel fluctuation. The significantly lower values of the last 4.85–5 ns (purple) of the process, however, clearly indicate that in the presence of atrazine, the fluctuation of the residues varies between states with little structural differences.

**Figure 4:**
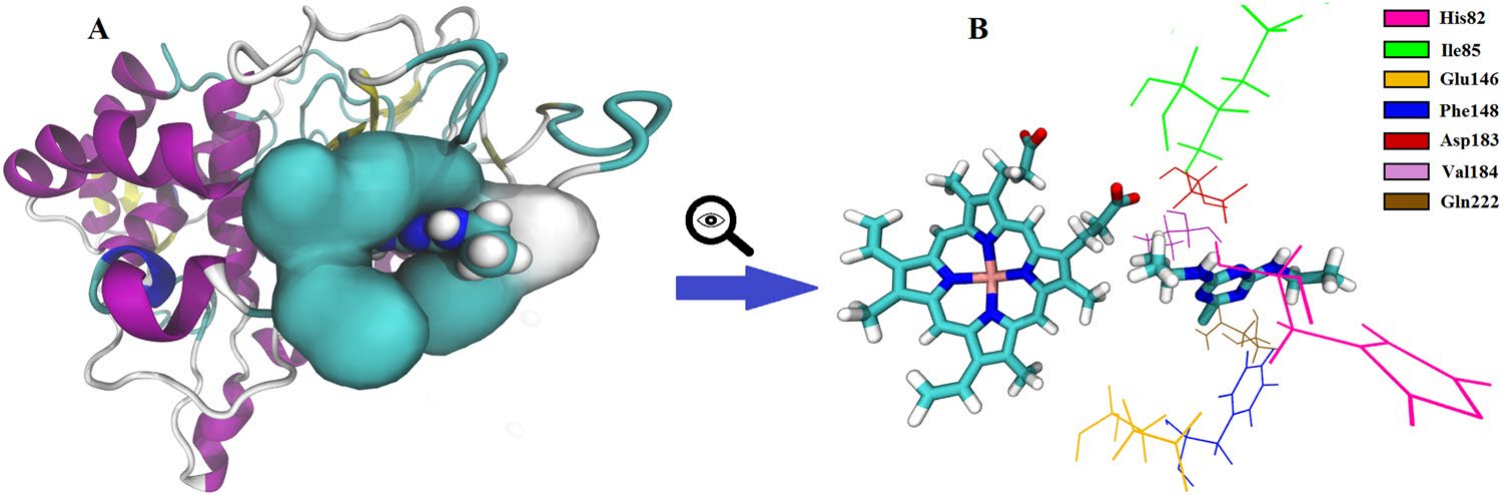
The final state of the [298 K] lignin peroxidase-atrazine complex after a 5 ns MD simulation. (**A**) The atrazine is stably integrated into the ligand channel residues, therefore, it cannot get through the channel. The residues forming the access channel are highlighted with QuickSurf representation. The resolution of the ligand channel residues was intentionally reduced to emphasize the position of the ligand between the residues. (**B**) The isopropylamine side chain of atrazine is the only group of the ligand which reaches through the channel, so atrazine cannot interact with the heme. The atrazine and heme are shown in Licorice representation. Coloring is based on atom types. The seven ligand channel residues are represented with coloured lines.

In the presence of atrazine, the channel fluctuation decreases in a short time, and the last 150 ps of the refining (5 ns) MD simulation shows significantly lower RMSD values compared to the data obtained from various timescales (Fig. 3). Since the conformational changes of the ligand channel residues in the absence of atrazine were clearly detected (Supplementary Fig. S1), the first 150 ps of the MD simulation without atrazine was used as a reference. In the presence of atrazine, the first 150 ps of the refinement shows little differences, and the 151–300 ps interval values are clearly lower than the reference in the whole range. After approximately 80 ps, those changes remain mostly under the values of the first 150 ps (Supplementary Table S2). The visualization of the lignin peroxidase-atrazine complex final state (Fig. 4) confirms that the atrazine molecule does not reach the active site through the channel because the ligand is surrounded by the mildly fluctuating ligand channel residues (Fig. 4A). This causes steric hindrance of the active site from the ligand. The shrinkage of the ligand channel residues occurs in a short time (ps scale) in the presence of atrazine, and this mild fluctuation is stably maintained over 4–5 ns (Supplementary video). The relative position of atrazine and the heme group (Fig. 4B) indicates that only the hydrophobic isopropylamine group of atrazine and some of the heme side chains are oriented towards each other.

Substrates can be oxidized via an electron transfer mediated by the lignin peroxidase surface residue Trp171, a hypothesis based on the observation that the mutation of this residue led to the loss of enzymatic activity toward veratrol oxidation. But mutants were still able to perform the oxidation of two dye substrates, 2,2′-azinobis(3-ethylbenzothiazoline-6-sulfonate) and 4-[(3,5-difluoro-4-hydroxyphenyl)azo]benzenesulfonic acid that had a lower redox potential compared to veratrol such as atrazine^23,24,29^. In the absence of Trp171, lignin peroxidase is still able to catalyze reactions, but a direct contact between heme edge and ligand through the ligand channel is essential. The exact function of Trp171 is not fully understood since this is a rigid area of lignin peroxidase, and a W171A mutation does not produce any relevant structural change^25^. Based on its rigid character, it is unlikely that the Trp171 area can function as a competitive ligand access channel.

The energy data (Supplementary Fig. S3) suggests that the reduced fluctuation of the ligand channel residues in the presence of atrazine is a result of favourable interactions and stable contact between the residues and the ligand. The energy results of the last 150 ps of the 5 ns long simulation (Supplementary Fig. S3) show that the complex is in equilibrium state, energy values are negative in the whole range (Supplementary Table S4).

The calculated binding free energies (Δ*G*_bin_) confirm atrazine can integrate into the environment of the ligand channel, however, the last 150 ps of MD show the slowly rising trend of binding free energies. Besides, the Δ*G*_bin_ between atrazine and heme have a positive value at certain times; these results indicate unfavourable and temporary contacts (Supplementary Fig. S5).

The additional 100-ns-long MD simulaton shows that after 5.6–5.7 ns the contacts between atrazine and the ligand channel start to break up. The visualization and RMSD values of the simulation clearly indicate that the integration of the ligand is only a temporary phenomenon. The partial atomic charges of atrazine create a temporary ligand-access channel complex but the kinetic energy of the enzyme along with unfavourable interactions coming from some heme side chain atoms interacting with the isopropylamine group of atrazine can overcome these effects in a short time, therefore, atrazine cannot reach the heme edge. Atrazine can be displaced from the ligand access channel, and the enzyme returns to its original state with fluctuating ligand channel residues, and periodic changes of open and closed states (Supplementary Fig. S6). During 100 ns, secondary structure of the enzyme has not been changed significantly.

Three of the ligand channel residues have the most significant impact on atrazine’s inaccessibility: Phe148, Asp183 and Gln222. The Phe148’s phenyl group interacts with the s-triazine group’s delocalized electron system. The carbon atom of Asp183’s carboxyl group has a partial charge of 0.62, and interacts with s-triazine’s N1 atom with a partial charge of −0.759. The hydrogens on Gln222’s amino group have a partial charge of 0.32 and 0.30, respectively. These atoms interact with s-triazine ring’s N1, N2, N3 and Cl atoms with partial charge of −0.759, −0.244, −0.19 and −0.26, respectively.

It is important to note that the previously calculated partial atomic charges of the carbons and nitrogen number 1 in the delocalized s-triazine ring of atrazine have significantly higher absolute values compared to the other atoms of the compound (C1: 0.405, C2: 0.401, C3: 0.483, N1: −0.759)^37^. The position of the incorporated atrazine (Fig. 4) suggests that these partial charges generated by the delocalized electron system are possible limiting factors, and could be responsible for the inaccessibility of the ligand.

### Conclusions of the results

This work reports the MD study of the interactions between the ligand channel residues of lignin peroxidase exoenzyme and atrazine herbicide. The enzyme is capable of degrading various xenobiotics, but the enzyme-substrate contacts necessary for catalysis are not fully understood. Since lignin peroxidase cannot degrade atrazine, a description of the interactions at the molecular level can give a better understanding of the structure-function relations of the ligand access channel and the factors that define or restrict the accessibility of certain ligands. Based on the results of protein-ligand docking, atrazine can find an energetically favourable position in the environment of the ligand channel residues, although, the mathematical probability of the formation of these complexes is not significant (10%, 16% and 53% frequency values at three different temperatures). During the 5 ns MD simulation of the atrazine-lignin peroxidase complex with the best docking result (53% rate at 298 K temperature), atrazine integrated into the residues and was not able to reach through the channel. This phenomenon is somewhat contradictory to that small substrates have clear access towards the heme^25^ when the channel is in an open state conformation^26^. The partial atomic charges of the delocalized heterocyclic s-triazine ring of atrazine can create a stable protein-ligand complex, and this could be the explanation of the inaccessibility of atrazine.

Since atrazine can bind to the residues that should give access for substrates towards the active site, it is an important question whether this binding has a temporary impact on enzyme activity. If atrazine can act as an inhibitor for individual substrates, such experimental results would prove that our theoretical approach describes the phenomenon correctly, therefore, this is our scope for further studies. Our results suggest that kinetic energy of the atoms can even expel atrazine from the channel, this possible phenomenon could be a scope of an other study. Besides, the exact function of the ligand access channel could be explained. Note that lignin peroxidase has two oxidation sites^23,24^, but the connection between them is still unclear.

## Electronic supplementary material

Supplementary Information

Supplementary Video
