# Supplementary material for: Lignin peroxidase ligand access channel dysfunction in the presence of atrazine
Source: Sci Rep. 2018 Apr 16;8:5989. doi: 10.1038/s41598-018-24478-w (PMC5902622; doi:10.1038/s41598-018-24478-w)
Supplement: Supplementary file 1 — Supplementary Information [file 41598_2018_24478_MOESM1_ESM.pdf]

Lignin peroxidase ligand access channel dysfunction in the presence of atrazine

János Ecker[1] and László Fülöp[1,*]

[1] Szent István University, Department of Chemistry, 2100, Gödöllő, Hungary
[*] Dr.Fulop.Laszlo@gmail.com

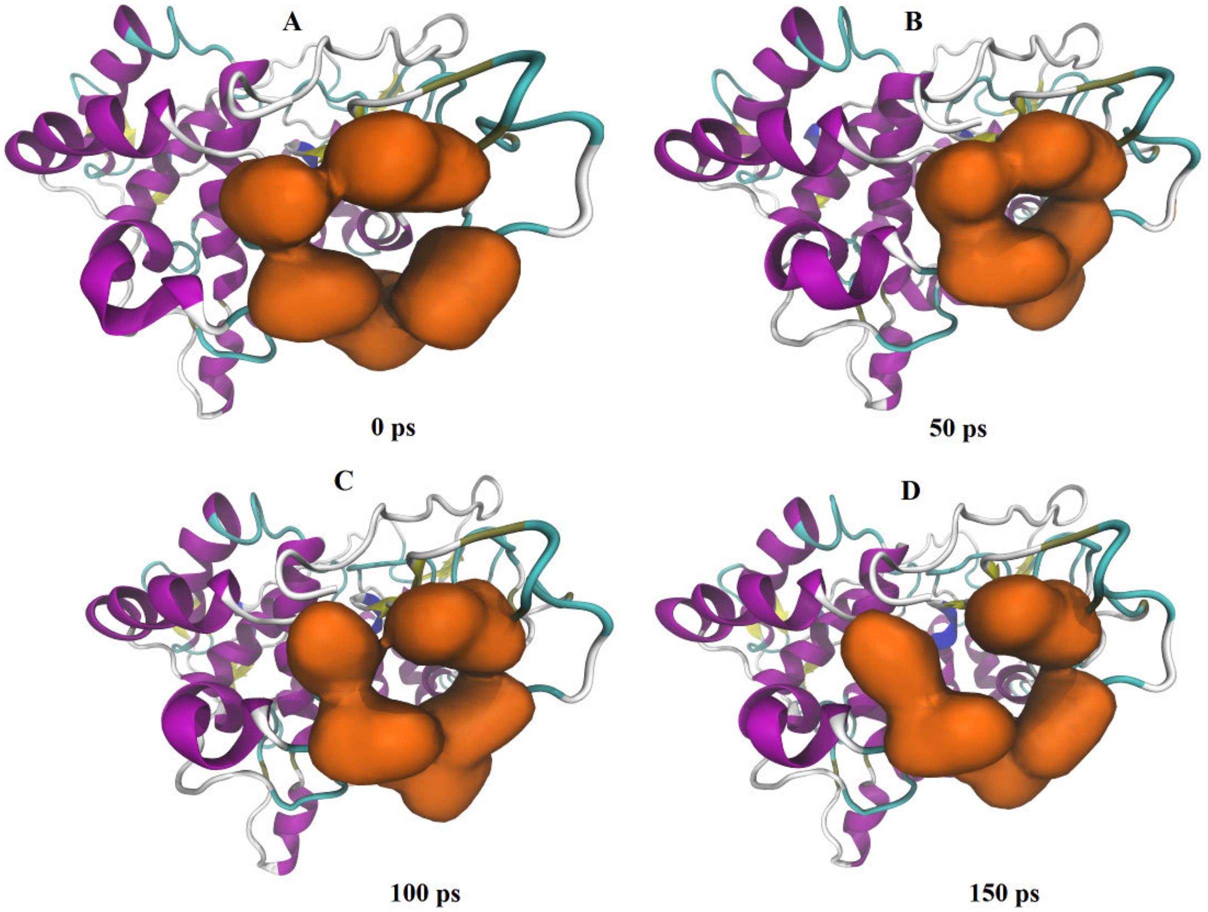

Supplementary Figure S1. Structural changes of the ligand channel residues of lignin peroxidase during a 150-ps-long MD simulation. The starting point (A) is the solvated and minimized crystal structure of lignin peroxidase. The structural difference is visible after 50 ps (B); residues are closer to each other, but at 150 ps (D), the state of the channel is comparable to the starting structure. The change taking place between A and B can be interpreted as a start of a closing sequence, and C-D states are moments from the continuous opening sequence. The residues forming the access channel are shown in dark orange with QuickSurf representation. The resolution of the ligand channel residues was intentionally reduced to emphasize the conformational changes between the four states.

frame	mol0	mol1	mol2	mol3
0	1.946	1.879	1.584	0.385
1	1.79	1.555	1.68	0.482
2	1.779	1.521	1.629	0.496
3	1.677	1.595	1.621	0.512
4	1.844	1.471	1.718	0.487
5	1.75	1.321	1.66	0.461
6	1.898	1.214	1.628	0.431
7	1.699	1.507	1.542	0.406
8	1.822	1.261	1.508	0.496
9	1.674	1.31	1.74	0.589
10	1.649	1.226	1.556	0.601
11	1.729	1.179	1.57	0.47
12	1.765	1.201	1.591	0.692
13	1.69	1.321	1.578	0.571
14	1.761	1.396	1.549	0.469
15	1.749	1.442	1.592	0.6
16	1.673	1.33	1.627	0.521
17	1.685	1.407	1.755	0.693
18	1.732	1.317	1.65	0.657
19	1.759	1.36	1.636	0.593
20	1.653	1.638	1.548	0.67
21	1.641	1.565	1.592	0.667
22	1.722	1.475	1.72	0.614
23	1.913	1.46	1.782	0.554
24	1.708	1.574	1.732	0.603
25	1.818	1.599	1.773	0.65
26	1.657	1.578	1.756	0.427
27	1.911	1.531	1.633	0.598
28	1.684	1.432	1.565	0.521
29	1.748	1.563	1.601	0.478
30	1.784	1.739	1.6	0.598
31	1.874	1.648	1.784	0.523
32	1.887	1.552	1.663	0.572
33	1.836	1.487	1.807	0.471
34	1.858	1.401	1.508	0.465
35	1.958	1.422	1.757	0.687
36	1.863	1.345	1.697	0.531
37	1.941	1.427	1.831	0.559
38	1.768	1.42	1.695	0.571
39	1.9	1.512	1.789	0.461
40	1.834	1.53	1.758	0.515
41	1.686	1.543	1.692	0.526
42	1.805	1.546	1.741	0.548
43	1.773	1.609	1.815	0.598

frame	mol0	mol1	mol2	mol3
75	1.984	1.392	1.311	0.643
76	1.941	1.464	1.612	0.563
77	1.916	1.214	1.606	0.556
78	2.004	1.298	1.623	0.531
79	1.935	1.451	1.619	0.527
80	1.911	1.556	1.481	0.431
81	1.967	1.571	1.687	0.676
82	1.937	1.623	1.596	0.521
83	1.999	1.644	1.582	0.524
84	1.845	1.687	1.612	0.449
85	1.828	1.403	1.598	0.412
86	1.873	1.506	1.453	0.559
87	1.873	1.617	1.45	0.525
88	1.72	1.603	1.405	0.488
89	1.784	1.518	1.492	0.637
90	1.783	1.531	1.488	0.537
91	1.809	1.625	1.513	0.489
92	1.915	1.641	1.372	0.622
93	1.709	1.67	1.535	0.668
94	1.937	1.503	1.496	0.587
95	1.89	1.661	1.49	0.536
96	1.616	1.588	1.425	0.565
97	1.832	1.516	1.347	0.593
98	1.674	1.679	1.571	0.696
99	1.806	1.548	1.573	0.76
100	1.695	1.584	1.518	0.74
101	1.626	1.468	1.585	0.782
102	1.794	1.499	1.469	0.717
103	1.863	1.344	1.423	0.598
104	1.692	1.425	1.68	0.554
105	1.885	1.637	1.514	0.609
106	1.719	1.582	1.505	0.605
107	1.902	1.572	1.496	0.62
108	1.979	1.644	1.584	0.555
109	1.901	1.654	1.618	0.672
110	1.91	1.734	1.527	0.779
111	1.889	1.679	1.447	0.642
112	1.911	1.633	1.52	0.701
113	1.872	1.508	1.571	0.734
114	1.736	1.502	1.471	0.815
115	1.62	1.62	1.447	0.787
116	1.731	1.652	1.537	0.924
117	1.843	1.643	1.474	0.644
118	1.883	1.797	1.479	0.674

44	1.889	1.514	1.779	0.559	119	1.833	1.668	1.279	0.948
45	1.884	1.59	1.819	0.768	120	1.759	1.717	1.473	0.701
46	1.725	1.68	1.733	0.75	121	1.775	1.665	1.596	0.737
47	1.883	1.713	1.645	0.676	122	1.718	1.698	1.461	0.788
48	1.889	1.48	1.88	0.703	123	1.627	1.676	1.535	0.882
49	1.827	1.642	1.674	0.628	124	1.758	1.675	1.451	0.957
50	1.983	1.459	1.628	0.556	125	1.727	1.61	1.455	0.809
51	2.076	1.634	1.758	0.478	126	1.711	1.719	1.496	0.712
52	2.012	1.565	1.659	0.587	127	1.742	1.643	1.395	0.516
53	2.054	1.59	1.673	0.513	128	1.841	1.592	1.553	0.687
54	1.958	1.474	1.856	0.514	129	1.873	1.699	1.533	0.751
55	1.864	1.5	1.847	0.33	130	1.804	1.811	1.488	0.749
56	2.004	1.397	1.687	0.449	131	1.815	1.585	1.6	0.607
57	2.107	1.391	1.544	0.495	132	1.746	1.607	1.421	0.737
58	1.968	1.645	1.58	0.533	133	1.828	1.618	1.601	0.696
59	2.012	1.396	1.527	0.56	134	1.806	1.756	1.564	0.679
60	1.93	1.438	1.77	0.592	135	1.85	1.556	1.463	0.778
61	1.897	1.412	1.703	0.625	136	2.07	1.694	1.557	0.772
62	1.919	1.487	1.738	0.577	137	1.964	1.791	1.582	0.621
63	1.935	1.537	1.538	0.567	138	1.931	1.551	1.49	0.767
64	1.779	1.459	1.627	0.49	139	1.971	1.645	1.494	0.778
65	1.864	1.338	1.711	0.557	140	1.79	1.74	1.414	0.794
66	1.816	1.422	1.777	0.631	141	1.817	1.809	1.535	0.758
67	1.895	1.513	1.78	0.506	142	1.896	1.843	1.407	0.82
68	1.933	1.605	1.689	0.464	143	1.973	1.708	1.552	0.71
69	1.978	1.497	1.769	0.499	144	1.818	1.639	1.619	0.719
70	1.975	1.6	1.724	0.631	145	1.78	1.678	1.533	0.784
71	1.933	1.619	1.713	0.698	146	1.687	1.604	1.539	0.696
72	1.997	1.665	1.621	0.585	147	1.718	1.444	1.372	0.775
73	2.024	1.339	1.534	0.65	148	1.773	1.693	1.396	0.757
74	1.911	1.422	1.425	0.503					

Supplementary Table S2. Fluctuation of the ligand channel in the presence of atrazine on various timescales with the exact values.

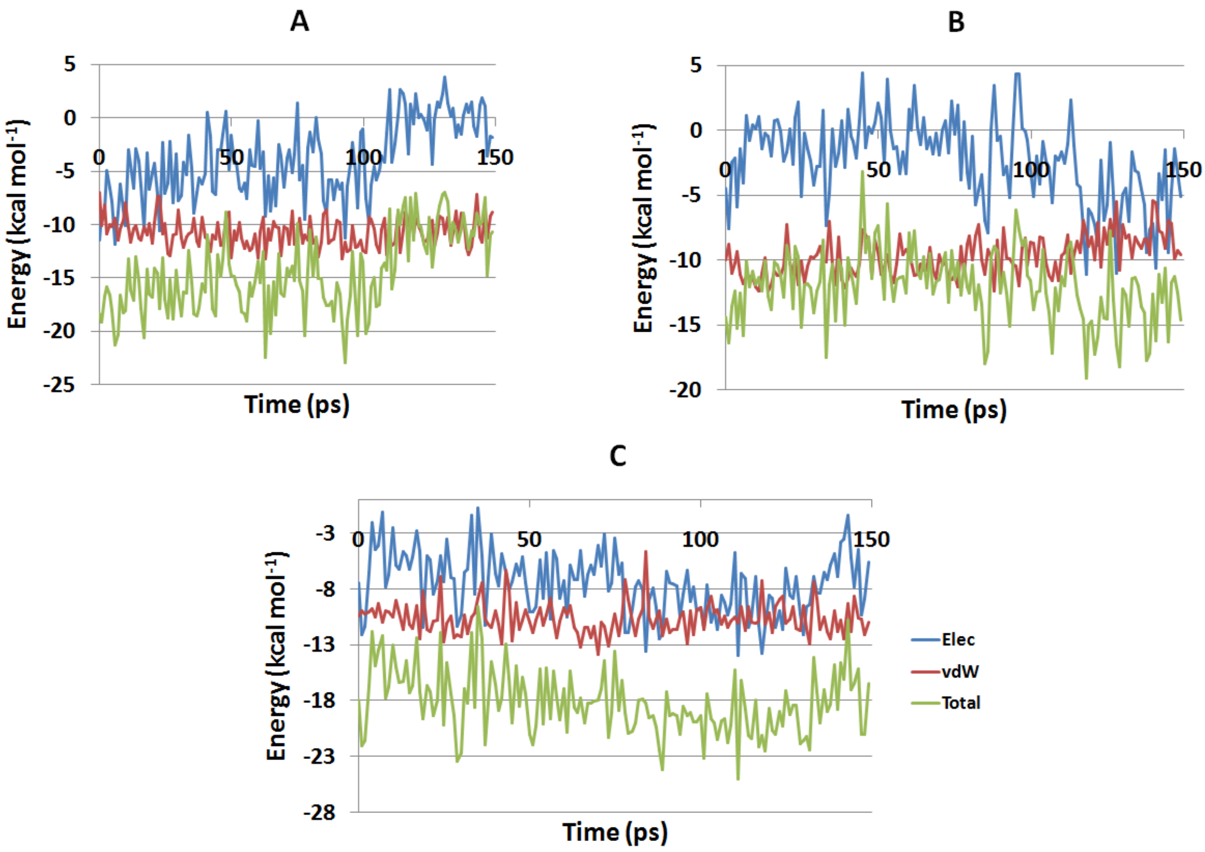

Supplementary Figure S3. The energy content of non-bonded interactions (kcal mol^{-1}) of the ligand channel residues-atrazine complex at various timescales. The sum of the electrostatic (blue) and van der Waals (red) energies represents the total non-bonded energy (green) of the complex. It is evident that directly after the minimization in the 0-150 and 150-300 ps intervals (A, B), the system has a higher energy content, the last 150 ps of the 5 ns MD (C) shows that the system has reached a stable, equilibrated state.

Time	Elec	VdW	Total
0	-7.4709	-10.4314	-17.9023
1	-12.1281	-9.9431	-22.0712
2	-11.3422	-10.1933	-21.5355
3	-6.5391	-10.0846	-16.6237
4	-2.0955	-9.7358	-11.8313
5	-4.4512	-10.4673	-14.9185
6	-4.1313	-9.3472	-13.4785
7	-1.1675	-11.0039	-12.1714
8	-7.815	-9.9521	-17.7671
9	-6.6544	-10.039	-16.6934
10	-2.5587	-10.5044	-13.0631
11	-5.903	-9.0266	-14.9296
12	-6.2229	-10.1595	-16.3824
13	-4.6935	-11.5896	-16.2831
14	-5.0447	-9.4102	-14.4549
15	-6.1955	-11.1415	-17.337
16	-5.1193	-11.5211	-16.6404
17	-2.8444	-9.4922	-12.3366
18	-4.6984	-12.459	-17.1574
19	-11.4236	-8.1752	-19.5988
20	-5.0097	-11.6048	-16.6145
21	-5.4115	-11.8309	-17.2424
22	-8.4511	-10.8729	-19.324
23	-7.2112	-10.7655	-17.9767
24	-5.0463	-6.8555	-11.9018
25	-7.3904	-12.7418	-20.1322
26	-3.5035	-11.1101	-14.6136
27	-6.9493	-10.4138	-17.3631
28	-7.0559	-12.3234	-19.3793
29	-11.3054	-12.0901	-23.3955
30	-10.3689	-12.2897	-22.6586
31	-6.5283	-10.3618	-16.8901
32	-6.1931	-12.0266	-18.2197
33	-1.4433	-10.4695	-11.9128
34	-8.4571	-10.1784	-18.6355
35	-0.7545	-8.8154	-9.5699
36	-5.0404	-7.4067	-12.4471
37	-11.2095	-10.6896	-21.8991
38	-7	-11.1475	-18.1475
39	-3.0619	-11.4425	-14.5044
40	-6.6039	-9.9841	-16.588
41	-7.7375	-11.1625	-18.9
42	-4.8664	-12.8925	-17.7589
43	-6.521	-6.3684	-12.8894

Time	Elec	VdW	Total
75	-3.4239	-10.1911	-13.615
76	-6.6767	-12.2073	-18.884
77	-5.6607	-10.4795	-16.1402
78	-11.9264	-7.1663	-19.0927
79	-11.9268	-8.9503	-20.8771
80	-9.7965	-10.9197	-20.7162
81	-7.8178	-12.1543	-19.9721
82	-7.2377	-10.68	-17.9177
83	-8.0786	-9.8092	-17.8878
84	-13.5424	-4.6653	-18.2077
85	-8.9867	-10.535	-19.5217
86	-7.8642	-11.5144	-19.3786
87	-9.669	-10.7121	-20.3811
88	-12.5061	-9.9365	-22.4426
89	-11.9688	-12.1531	-24.1219
90	-6.4641	-10.6992	-17.1633
91	-7.4763	-11.8597	-19.336
92	-7.494	-11.6435	-19.1375
93	-7.6899	-11.609	-19.2989
94	-10.5145	-10.537	-21.0515
95	-8.4552	-10.0281	-18.4833
96	-6.356	-12.9454	-19.3014
97	-9.0963	-9.9634	-19.0597
98	-7.8478	-12.0747	-19.9225
99	-9.9772	-9.8999	-19.8771
100	-9.6371	-9.7095	-19.3466
101	-11.4764	-11.6455	-23.1219
102	-7.5937	-9.7733	-17.367
103	-10.9488	-8.6576	-19.6064
104	-9.8183	-10.1477	-19.966
105	-11.5968	-9.8863	-21.4831
106	-8.6011	-11.1823	-19.7834
107	-9.2047	-10.4438	-19.6485
108	-10.3115	-11.4048	-21.7163
109	-9.26	-10.8889	-20.1489
110	-4.7329	-10.5073	-15.2402
111	-13.9487	-11.0568	-25.0055
112	-6.5886	-9.5939	-16.1825
113	-7.0816	-11.7385	-18.8201
114	-11.4705	-9.594	-21.0645
115	-10.3713	-10.9781	-21.3494
116	-6.9008	-11.0284	-17.9292
117	-11.2625	-10.8588	-22.1213
118	-13.758	-7.3037	-21.0617

44	-8.0931	-7.9349	-16.028	119	-11.3468	-11.1366	-22.4834
45	-7.3767	-12.6073	-19.984	120	-8.5373	-10.1533	-18.6906
46	-5.7253	-9.1793	-14.9046	121	-8.8116	-11.8598	-20.6714
47	-6.8341	-11.6663	-18.5004	122	-11.4358	-9.5899	-21.0257
48	-5.1539	-10.7587	-15.9126	123	-9.959	-8.9586	-18.9176
49	-7.4888	-9.8835	-17.3723	124	-11.6204	-8.6735	-20.2939
50	-10.0156	-10.9487	-20.9643	125	-6.1156	-11.0254	-17.141
51	-10.0443	-11.8678	-21.9121	126	-8.5671	-10.7063	-19.2734
52	-9.5598	-10.7476	-20.3074	127	-8.7926	-9.5851	-18.3777
53	-5.4059	-9.7129	-15.1188	128	-6.8628	-11.5302	-18.393
54	-8.8439	-11.2322	-20.0761	129	-10.1897	-11.6855	-21.8752
55	-4.7586	-10.333	-15.0916	130	-12.0794	-9.5288	-21.6082
56	-10.7183	-8.8484	-19.5667	131	-9.6109	-11.5393	-21.1502
57	-4.5875	-10.794	-15.3815	132	-9.4206	-12.9441	-22.3647
58	-5.34	-12.3408	-17.6808	133	-6.8431	-7.3044	-14.1475
59	-8.7946	-10.8806	-19.6752	134	-8.3945	-8.8328	-17.2273
60	-7.2243	-9.6683	-16.8926	135	-8.3868	-11.5936	-19.9804
61	-10.507	-10.3235	-20.8305	136	-6.4746	-10.5524	-17.027
62	-5.8676	-9.4671	-15.3347	137	-7.1736	-11.812	-18.9856
63	-7.1637	-11.4385	-18.6022	138	-5.9053	-12.4532	-18.3585
64	-7.1314	-11.924	-19.0554	139	-4.8326	-9.9526	-14.7852
65	-4.5241	-13.1945	-17.7186	140	-6.9253	-11.8214	-18.7467
66	-8.6095	-11.3977	-20.0072	141	-3.8519	-10.7806	-14.6325
67	-6.738	-11.4809	-18.2189	142	-3.5413	-12.4961	-16.0374
68	-5.8486	-12.3812	-18.2298	143	-1.4389	-9.3447	-10.7836
69	-6.6619	-11.3502	-18.0121	144	-5.2442	-11.7935	-17.0377
70	-4.1002	-13.8829	-17.9831	145	-7.8197	-8.6673	-16.487
71	-5.9729	-10.9837	-16.9566	146	-4.4768	-10.6413	-15.1181
72	-3.0458	-11.3534	-14.3992	147	-10.3015	-10.6956	-20.9971
73	-8.2154	-13.0744	-21.2898	148	-8.8793	-12.1322	-21.0115
74	-7.4441	-11.6301	-19.0742	149	-5.5618	-10.9361	-16.4979

Supplementary Table S4. The energy content of non-bonded interactions (kcal mol^{-1}) of the ligand channel residues-atrazine complex at various timescales with the exact values.

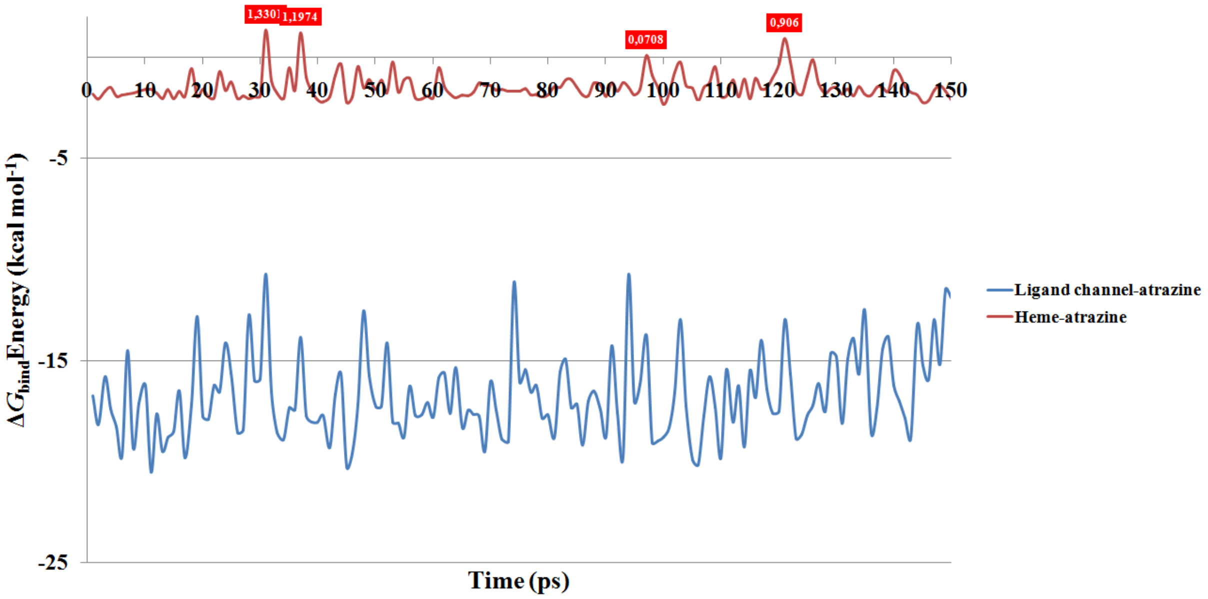

Supplementary Figure S5. The binding between atrazine and ligand channel (blue) residues is favourable. Through the calculation of atrazine-heme binding free energies (red), several positive values were detected (the values are represented) which suggests unfavourable, temporary contacts.

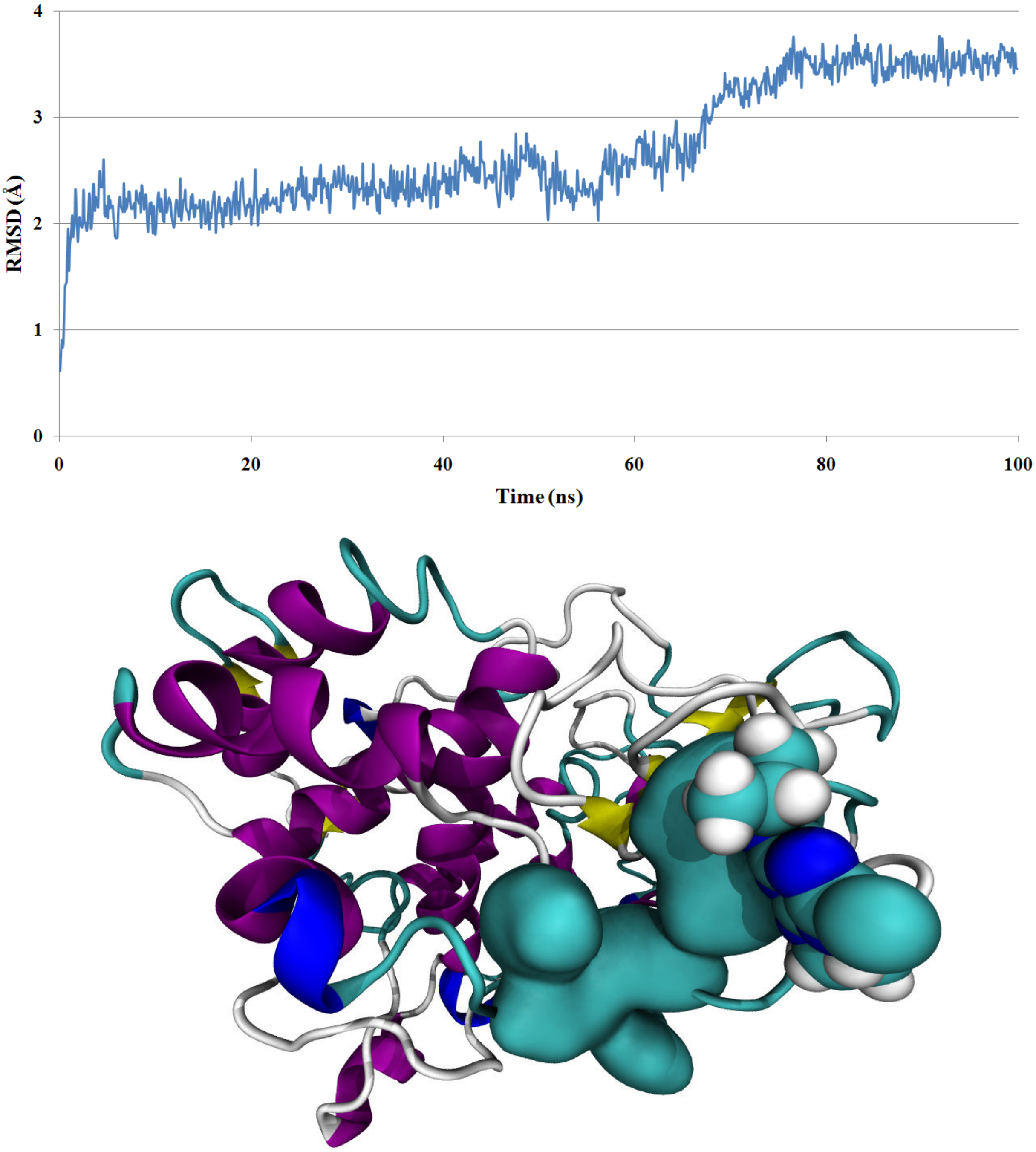

Supplementary Figure S6. The RMSD values (up) of the additional 100 ns MD simulation clearly show that the kinetic energy of the ligand channel residues can overcome the stabilizing effect of atrazine's partial atomic charges. After the atrazine is removed from the channel, the channel's fluctuation rapidly increases, and the enzyme returns to its original state. The final state (down) of the atrazine is visualized.
